# Computer-Assisted Cognitive-Behavioral Therapy to Treat Adolescents With Depression in Primary Health Care Centers in Santiago, Chile: A Randomized Controlled Trial

**DOI:** 10.3389/fpsyt.2019.00552

**Published:** 2019-07-30

**Authors:** Vania Martínez, Graciela Rojas, Pablo Martínez, Jorge Gaete, Pedro Zitko, Paul A. Vöhringer, Ricardo Araya

**Affiliations:** ^1^Centro de Medicina Reproductiva y Desarrollo Integral del Adolescente (CEMERA), Faculty of Medicine, Universidad de Chile, Santiago, Chile; ^2^Millennium Nucleus to Improve the Mental Health of Adolescents and Youths (Imhay), Santiago, Chile; ^3^Millennium Institute for Depression and Personality Research (MIDAP), Santiago, Chile; ^4^Department of Psychiatry and Mental Health, Clinical Hospital, Universidad de Chile, Santiago, Chile; ^5^School of Psychology, Faculty of Humanities, Universidad de Santiago de Chile, Santiago, Chile; ^6^Department of Public Health and Epidemiology, Faculty of Medicine, Universidad de Los Andes, Santiago, Chile; ^7^Health Service & Population Research Department, Institute of Psychiatry, Psychology & Neuroscience, King’s College London, London, United Kingdom; ^8^Unit of Healthcare Studies, Complejo Asistencial Barros Luco, Santiago, Chile; ^9^Mood Disorders Program, Tufts Medical Center, Tufts University, Boston, MA, United States

**Keywords:** depression, adolescent, cognitive therapy, computer-assisted therapy, primary health care, randomized controlled trial

## Abstract

**Introduction:** Evidence from developed countries shows the efficacy of computer-assisted cognitive-behavioral therapy (cCBT) in addressing adolescent depression in home and/or school settings. This paper presents the results of a randomized controlled trial (RCT) of a brief therapist-guided cCBT intervention for adolescent depression in resource-constrained primary health care (PHC) settings.

**Material and methods:** A multicenter, two-arm parallel-group, individually RCT with a 1:1 allocation ratio assigned 216 depressed adolescents (aged 15–19) attending four PHC centers in a low-income municipality of Santiago, Chile, to receive eight weekly face-to-face therapist-guided cCBT sessions by study therapists (N = 108), or to receive an enhanced usual care (EUC) intervention by trained PHC psychologists, encouraged to adhere to the national clinical guidelines for the management of depression (N = 108). Both groups received pharmacotherapy concordant with these guidelines. The primary outcome was the Beck Depression Inventory (BDI) at 4 months post-randomization, to assess depressive symptoms. BDI at 6 months post-randomization was a secondary outcome. Additional measures included patients’ compliance, and satisfaction with different treatment components, at 6 months post-randomization.

**Main Results:** The adjusted difference in mean BDI score between groups was -3.75 (95% CI -6.23 to -1.28; p = 0.003) at 4 months post-randomization. At 6 months post-randomization, the adjusted difference in mean BDI score between groups was -2.31 (95% CI -4.89 to 0.27; p = 0.078). The effect size was small-to-medium at 4 months post-randomization, d = 0.39 (0.12 to 0.67), and small and non-significant at 6 months post-randomization d = 0.29 (-0.00 to 0.59). Adolescents in the experimental treatment group were significantly more satisfied with treatment, with the PHC centers’ facilities, with the psychological care received, and with non-professional staff than those in the comparator treatment group.

**Discussion:** A brief therapist-guided cCBT eight-session intervention improves the response of depressed adolescents attending PHC centers at 4 months post-randomization. At 6 months post-randomization, the differences of between groups were not significant. Future research may focus on exploring strategies to sustain and increase response.

**Clinical trial registration:** www.ClinicalTrials.gov, identifier NCT01862913 and URL: https://clinicaltrials.gov/ct2/show/NCT01862913.

## Introduction

### Adolescent Depression: A Global Health Concern

The pooled prevalence of major depressive disorders (MDD) among adolescents is estimated at 4% to 5% (1), with higher rates found in vulnerable populations (2). The severity of depressive symptoms among adolescents has been associated with suicidal ideation (3, 4), and subsequent alcohol and illicit drug use (5). In addition, adolescent depression has been linked to poor living standards and worse mental health in adulthood (6, 7). Thus, the effective management of depression in this population is a global health concern (8).

### Evidence-Based Interventions for Adolescent Depression

Studies have demonstrated that several effective interventions for addressing adolescent depression exist. Interpersonal and cognitive-behavioral psychotherapies (CBT) have shown to be efficacious and acceptable (9), while fluoxetine has demonstrated superior efficacy and tolerability amongst various antidepressants (10, 11). Combined treatment, involving medication and psychotherapy, has been recommended for moderate to severe cases (12, 13), as it is likely to be more cost-effective than stand-alone therapies, although the superiority of any of these evidence-based interventions (EBIs) remains inconclusive (14).

In addition to its effectiveness, patient preference may be an argument for the selection of psychotherapies as a promising treatment alternative for adolescent MDD (15), with CBT—aimed at changing dysfunctional thoughts and cognitive distortions (16)—being recognized as the dominant intervention model (17). However, a large proportion of depressed adolescents do not receive EBIs or receive no treatment at all (18, 19). Moreover, implementation of EBIs strongly relies on proper provider training and fidelity to intervention manuals (18, 20).

### Technology-Assisted Psychotherapies for Adolescent Depression

The use of digital technologies as an alternative or complement to conventional mental health services has the potential to increase treatment access for underserved populations (21, 22) and to promote the integration of EBIs into real-world adolescent settings (21, 23). Additionally, when carefully designed, technological interventions may be persuasive enough to warrant the attention of youth users, thus improving adherence, satisfaction, and clinical outcomes (24). Though promising, technology-assisted psychotherapies for adolescent depression are in an experimental phase (17).

Computer-assisted CBT (cCBT) has been one of the most studied technology-assisted interventions for youth mental health (25, 26). cCBT may be purely self- or therapist-guided, with the latter improving adherence and clinical outcomes (26). With demonstrated efficacy in home and/or school settings (25, 26), cCBT may enhance the integration of EBIs into primary health care (PHC). Moreover, if interactive enough, therapist-guided cCBT may be better accepted, providing an enhanced learning experience, and opportunities to tailor treatment to PHC patient’s needs.

### A cCBT for Adolescent Depression in PHC: The Chilean Context

Worldwide, evidence has highlighted the advantages of integrating child and adolescent mental health services into PHC (27, 28), which has been described as the ideal setting to provide first-contact health care, offering accessible, continuous, and family-centered care (29). Moreover, integration may help to reduce stigma, be acceptable and desirable by patients and clinicians, and be a unique opportunity to reach vulnerable populations (e.g., low-income, minority adolescents) (29). Chile, a developing nation, has not fallen behind in this respect.

During the past decades, important public mental health policies led to the integration of mental health into Chilean PHC (30), the paradigmatic example being the successful national scale-up of the Program of Treatment for Depression in Primary Health Care (PTD-PHC) for low- to middle-income individuals aged 15 and over (31). The PTD-PHC is a stepped-care program, involving PHC clinicians working in a coordinated fashion to provide a range of low-intensity psychosocial interventions with antidepressants, and treatment referral, if warranted (31).

In 2006, and after a major health care reform implemented within a social guarantee framework, depression was recognized as a national priority, ensuring financial protection and opportunity of access to the set of services included in the PTD-PHC for all people aged 15 and over (32). Additionally, the Chilean Ministry of Health Clinical Guidelines for the Management of Depression were used as important quality improvement tools for the management of depression in PHC (33).

Though promising, program evaluations have revealed that the effectiveness of the PTD-PHC is hindered by difficulties in making an accurate diagnosis of depression, and by low rates of treatment adherence (31, 34). Moreover, despite Chile having one of the highest prevalence of adolescent depression worldwide (7.0%) (35), there have been a dearth of research into adolescent depression treatment options in Chile. Furthermore, harnessing digital technologies within PHC settings may be particularly attractive to adolescents.

This paper presents the results of a randomized controlled trial (RCT) of a brief therapist-guided cCBT intervention for adolescent depression in resource-constrained PHC settings in Santiago, the capital city of Chile. Further details about the intervention and the study design have been published elsewhere (36).

## Materials and Methods

### Study Design

This was a multicenter, two-arm parallel-group, individually RCT with a 1:1 allocation ratio to either a nonpharmacological treatment intervention (therapist-guided cCBT) for adolescent depression in PHC or an enhanced usual care (EUC) intervention.

### Participants

Puente Alto is a low-income municipality of Santiago, Chile, with a large adolescent population. Adolescents aged 15–19 years detected as suspected of having depression by professionals from four PHC centers in Puente Alto or nearby schools were invited to participate in the study. The adolescents who consented were asked to answer the Beck Depression Inventory (BDI) (37). Adolescents with a score of at least 10 in the BDI were invited to a face-to-face semi-structured diagnostic interview, the Kiddie-SADS-Present and Lifetime Version (K-SADS-PL). The K-SADS-PL was administered by the principal author (VM, a certified child and adolescent psychiatrist), a properly trained psychologist [Marianela Hoffmann (MH)], or a certified child and adolescent psychiatrist with due training [Francesca Borghero (FB)], for the assessment of current and past DSM-IV psychopathology (38). Those adolescents meeting diagnostic criteria for depressive disorders, and not having a hypomania or mania, psychosis, or alcohol or substance dependence, were included. Additional exclusion criteria were: a) current suicidal risk requiring inpatient care or specialized management according to the Chilean Ministry of Health Clinical Guidelines for the Management of Depression (33); b) current antidepressant or psychological treatment; and c) low intellectual ability (i.e., the adolescent was attending to special education and/or had previous diagnosis).

### Interventions

Before the start of recruitment, physicians in the four participating PHC centers received a special training session of 2 h from VM, aimed at assisting with the correct identification, diagnosis, and treatment of adolescent patients with depression, according to the Chilean Ministry of Health Clinical Guidelines for the Management of Depression (33). This training was planned to ensure an adequate use of pharmacotherapy for adolescents with moderate to severe depression in the experimental and the comparator groups (i.e., if needed, starting fluoxetine at a daily dose of 10 mg, with titration to a maximum of 60 mg/day according to clinical response). Six months after the start of recruitment, a booster training session was offered.

#### Therapist-Guided cCBT Intervention

Adolescents allocated to the experimental treatment received a therapist-guided cCBT intervention called YPSA-M (for “Yo pienso, siento y actúo—mejor”; in English: “I think, feel, and behave—better”) plus EUC—as described in the Chilean Ministry of Health Clinical Guidelines for the Management of Depression (33). YPSA-M is a manualized cCBT intervention with the following components: a) psychoeducation for depression (symptoms, causes, and treatments), b) promotion of health lifestyles, c) promotion of adherence to medical treatment, d) behavioral activation techniques, e) presentation of the CBT model of depression (recognition of emotions, thoughts, behaviors, and their relationships), f) emotional regulation strategies, g) recognition and challenge of dysfunctional thoughts, h) recognition and reinforcement of support networks, and i) promotion of adaptive problem-solving attitudes and skills. Components of the intervention were presented to depressed adolescents through computer-based visually attractive and interactive examples, exercises, and videos.

This experimental treatment was delivered through weekly 40-min-long face-to-face individual sessions for a period of 8 weeks by study therapists at the PHC centers. A freely available version of the YPSA-M software was installed in the consulting room computers, with access restricted to study therapists (materials available upon request from the corresponding author). During sessions, therapists used the YPSA-M to deliver the intervention, which guided depressed adolescents on the use of the software and encouraged them to complete exercises and review personal cards with key learning points handed out after each session.

Study therapists were psychologists experienced in the treatment of depressed adolescents and with a basic understanding of CBT concepts. They received 12 h of training on the use of the intervention and were supervised by VM once a month and upon request (1 h). There was provided guidance to therapists on a wide range of clinical scenarios (e.g., how to proceed if the adolescent came to the session in a crisis situation, how to incorporate parents/guardians in the sessions).

Sessions were audio-recorded, and fidelity assessments were conducted to evaluate how closely the study therapists adhered to the cCBT intervention. The compliance was assessed using a verification checklist in a random sample of 10%.

Between sessions, the study coordinator contacted the participants to remind them of their appointments.

#### Enhanced Usual Care

Adolescents allocated to the comparator treatment received an EUC intervention provided by trained PHC psychologists in the four participating PHC centers. Psychologists received a special training session (2 h) from VM, aimed at assisting with the correct identification, diagnosis, and treatment of adolescent patients with depression, according to the Chilean Ministry of Health Clinical Guidelines for the Management of Depression (33). These guidelines emphasize that a psychosocial treatment plan for depression should incorporate the following: a) promotion of health lifestyles, regular physical activity, healthy diet, and sleep hygiene; b) psychoeducation to adolescents and their parents; and c) brief cognitive-behavioral or interpersonal-based interventions. Additionally, the treatment for adolescent depression should include pharmacological treatment, medical controls, and referral to secondary or tertiary psychiatric services, depending on the specificities of each case. This treatment, widely available in Chilean PHC centers, has been shown to be efficacious for depressed adults (39). Six months after the start of recruitment, a booster training session was offered.

### Outcomes

The primary outcome was the BDI score at 4 months post-randomization. The BDI is a well-established, psychometrically sound, 21-item self-report questionnaire used to assess key symptoms of depression (37). Items are scored on a 4-point scale (0–3) for a total score range of 0 to 63. Scores ranges of 0–9, 10–18, 19–29, and 30–63 are indicative of minimal, mild, moderate, and severe depressive symptoms, respectively. It has been previously used in Chile for the assessment of PHC depressed patients (31), and adolescents aged 13–19 years (40). The BDI provided a continuous score measured at baseline, and at 4 and 6 months post-randomization, with the latter follow-up period considered as a secondary outcome. In the study sample, the total Cronbach’s alpha (α) coefficient was 0.80 [95% confidence interval (CI) 0.67 to 0.84].

The secondary outcomes were defined as follows: a) the 10-item “personal failure” subscale (score range 0–40) of the Children’s Automatic Thoughts Scale (CATS) (41), a self-report scale measuring adolescent’ dysfunctional thoughts, with higher scores indicative of more severe dysfunctional thoughts; b) the Social Problem-Solving Inventory-Revised Short Form (SPSI-RS) (42), a 25-item self-report inventory (score range 0–20) assessing problem-solving abilities in adolescents, with higher scores representative of more effective problem-solving abilities; and c) the KIDSCREEN-27 item (five dimensions) health-related quality of life (HRQoL) questionnaire and the 10-item general index score (43), with higher scores characteristic of better HRQoL. This study used the Spanish versions of CATS and SPSI-RS, and the Chilean version of KIDSCREEN-27, which have been previously applied to Chilean adolescent population (40, 44, 45). These continuous outcome measures were assessed at baseline, and at 4 and 6 months post-randomization. In the study sample, α coefficients for these scales ranged from 0.86 to 0.90. Additional measures included patients’ compliance (i.e., attendance to at least five of eight sessions (46, 47), and satisfaction with different treatment components (with an *ad hoc* self-report questionnaire), at 6 months post-randomization. The proportion of participants who were prescribed antidepressants was also recorded as a control variable.

### Sample Size

It was anticipated that the therapist-guided cCBT intervention would produce a clinically meaningful improvement (remission, BDI score <10) in 60% of adolescents, and that only 40% of those in the comparator would reach this level of improvement in 4 months post-randomization. A difference of 20% would be regarded as clinically meaningful. To achieve a two-sided alpha coefficient of 5% and a statistical power of 80%, at least 97 adolescents were needed in each group to detect this difference, or 108 subjects per group, allowing for 10% attrition (39, 48). This total would yield an 80% power to detect a mean difference of at least 0.4 standard deviations in the primary outcome measure.

### Randomization and Blinding

Randomization was balanced using a block size of four and stratified by sex and severity of depression symptoms (mild, moderate, and severe), according to BDI score. Randomization was generated using web-based random allocation algorithms. Sequentially numbered, opaque, sealed envelopes were sent to each PHC center. VM, MH, and FB enrolled participants and opened sealed envelopes to determine treatment assignments. The outcomes assessors were not part of the baseline assessment and were blinded to treatment assignments.

### Data Analysis

The Consolidated Standards of Reporting Trials (CONSORT) statement was followed for reporting the results of this study (49). Descriptive analyses were carried out to assess the balance between the study groups. To deal with protocol deviations, all participants were included in the analysis regardless of adherence to the protocol (i.e., intention-to-treat approach) (49). Moreover, comparative analyses considered missing outcomes for some participants, with sensitivity analyses conducted to investigate the potential effects of missing data (50). In this regard, multiple imputations by chained equations were performed to deal with missing data (20 copies), after ensuring that data were missing at random (51). As results with and without imputed data were virtually the same, the main results were presented based only on complete case data. The primary analysis employed multivariable linear regression to investigate differences in mean BDI scores between groups at 4 months after randomization, adjusting for baseline BDI scores and sex. We also conducted a subgroup analyses for the primary outcome using interaction terms in the regression models between randomized arm (group) and the baseline depression scores categorized as mild, moderate, and severe. Effect sizes for BDI scores were estimated by calculating the standardized mean difference (Cohen’s *d*), with cutoffs of 0.20, 0.40, and 0.80 for small, medium, and large effect size, respectively (52). Similar analyses were conducted for the secondary outcomes. All analyses were performed using STATA 14.2.

### Protocol Registration

This study was registered as a clinical trial at clinicaltrials.gov, using the clinical trial unique identifier: NCT01862913; and URL: https://clinicaltrials.gov/ct2/show/NCT01862913.

## Results

### Sample and Participant Flow

**Figure 1:**
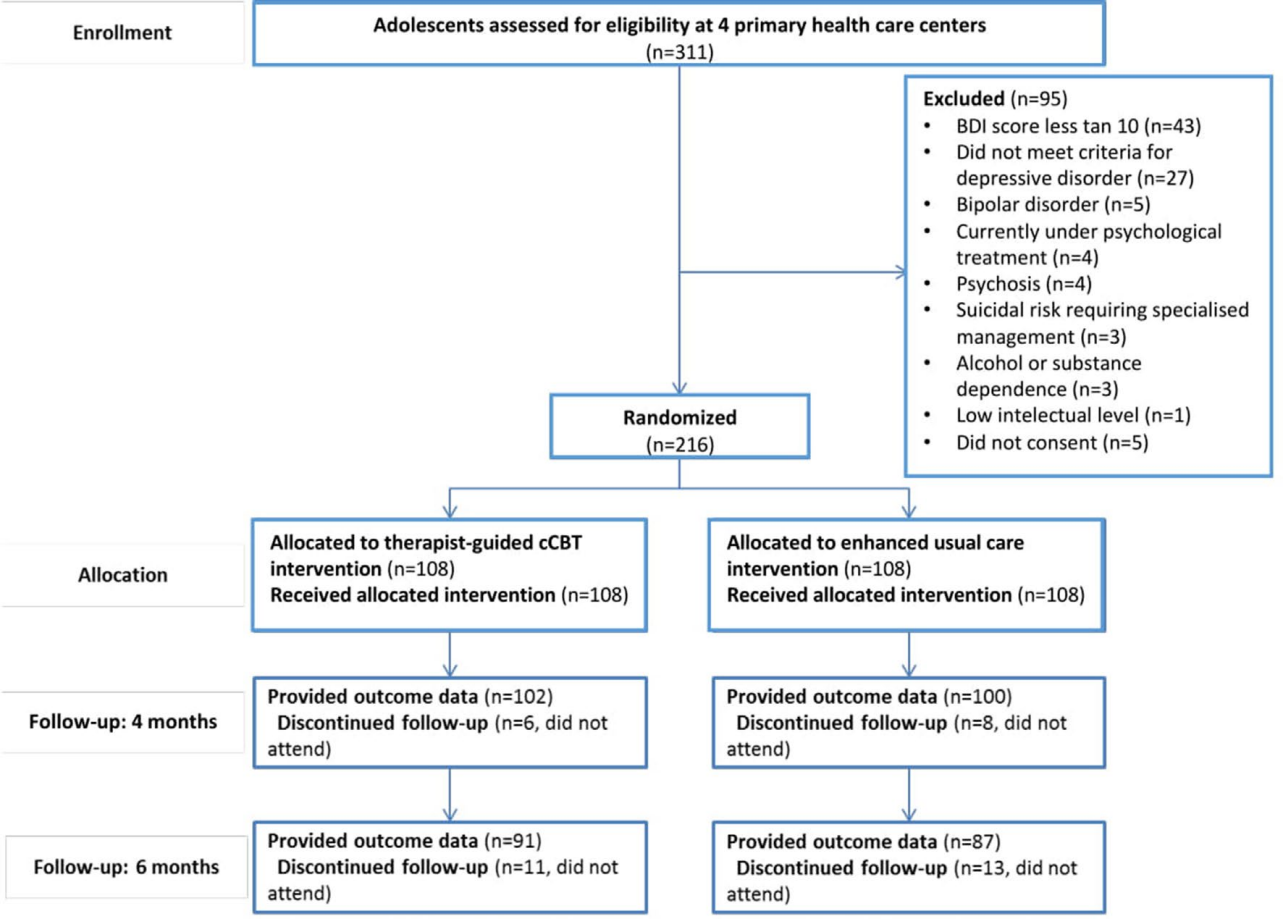
CONSORT flow diagram. Flow of participants.

A total of 311 adolescents were assessed for eligibility at the four PHC centers, but 90 did not meet eligibility criteria, and 5 did not consent to participate. Therefore, 216 adolescents were randomly allocated to either the therapist-guided cCBT intervention (experimental treatment group) or the EUC intervention (comparator treatment group). There were 202 (94%) participants who provided data for the primary outcome (4-month post-randomization BDI score), 93% in the experimental treatment *vs*. 94% in the comparator treatment (see **Figure 1**).

### Baseline Sample Characteristics

**Table 1** shows baseline characteristics for participants, most of whom came from low- or middle-socioeconomic household levels and were predominantly female, living with one or both parents. In addition, approximately 50% of participants had a personal history of receiving mental health assistance, while about 40% had a parental history of depression. The mean age of the sample at baseline was 16.3 years (SD = 1.1), with a mean education level of 9.2 years (SD = 1.4). The mean BDI score at baseline was 25.7 points (SD = 8.5) in the comparator treatment group and 25.5 points (SD = 7.9) in the experimental treatment group (see **Table 1**).

**Table 1:** Characteristics of participants at baseline by trial arm.

Characteristics	Therapist-guided cCBT intervention No. = 108	Enhanced usual care intervention No. = 108
Female sex, no. (%)	82 (75.9)	83 (76.9)
Age, mean (SD), y	16.2 (1.1)	16.3 (1.1)
Years of schooling, mean (SD), y	9.0 (1.3)	9.4 (1.4)
Living with, no. (%)		
Both parents	53 (49.07)	53 (49.07)
One of the parents	49 (45.37)	47 (43.52)
None of the parents	6 (5.56)	8 (7.41)
Socioeconomic level, no. (%)		
Low	47 (43.52)	47 (43.52)
Medium	60 (55.56)	59 (54.63)
High	1 (0.92)	2 (1.85)
Depressive symptom score, mean (SD)	25.7 (8.5)	25.5 (7.9)
Depressive symptoms severity, no. (%)		
Mild	28 (25.93)	25 (23.15)
Moderate	44 (40.74)	43 (39.81)
Severe	36 (33.33)	40 (37.04)
Personal history of mental health assistance, no. (%)	53 (49.07)	57 (52.78)
Parental history of depression, no. (%)	43 (39.82)	43 (39.82)

*cCBT, computer-assisted cognitive-behavioral therapy; No., frequency; SD, standard deviation; y, years.*

Depressive symptom score and severity according to Beck Depression Inventory.

### BDI Scores at 4 Months Post-Randomization

Statistically significant (adjusted) differences were found in the mean BDI scores between the two groups at 4 months post-randomization (-3.75, 95% CI -6.23 to -1.28, P = 0.003), with a small-to-medium effect size (*d* = 0.39, 95% CI 0.12 to 0.67). These differences favored the experimental treatment group (**Table 2**).

**Table 2:** BDI scores at 4 and 6 months post-randomization.

Analysis	Mean BDI score			*P* Value
	Therapist-guided cCBT intervention	Enhanced usual care intervention	Adjusted difference in means (95% CI)^a^	
4 months	13.2 (9.4)	17.1 (10.2)	–3.75 (-6.23 to -1.28)	0.003
No. (primary outcome)	100	102		
6 months	12.0 (9.2)	14.7 (9.9)	–2.31 (-4.89 to 0.27)	0.078
No.	87	91		

*BDI, Beck Depression Inventory; cCBT, computer-assisted cognitive-behavioral therapy; CI, confidence interval; No, frequency.*

Data are mean (standard deviation), unless otherwise specified.

a BDI score adjusted for sex and baseline BDI score.

### Secondary Outcomes

Adjusted differences in the mean BDI scores between the two groups at 6 months post-randomization favored the experimental treatment group, although this marginally failed to reach statistical significance -2.31 (95% CI -4.89 to 0.27, P = 0.078), and had a small (and non-significant) effect size (*d* = 0.29, -0.00 to 0.59) (**Table 2**). Complementarily, **Table 3** depicts the secondary outcomes (i.e., CATS, SPSI-RS, and KIDSCREEN-27) of the experimental treatment group *versus* the comparator treatment group, showing: a) statistically significant differences (P<0.05) in favor of the experimental treatment group at 4 months post-randomization consistent with lower levels of dysfunctional thoughts, better social problem-solving abilities, better physical and psychological well-being, and higher general HRQoL, achieved by the therapist-guided cCBT intervention group in comparison to the EUC intervention group; b) statistically significant differences (P = 0.009) in the school environment dimension of the KIDSCREEN-27 at 6 months post-randomization, favoring the experimental treatment group; c) with the exception of social problem-solving abilities, early significant differences between groups were not maintained at the 6 months post-randomization; and d) as for “autonomy and parent relation,” and “peers and social support” (two dimensions of the KIDSCREEN-27), no treatment group had a clear advantage over the other. In both groups, 51.85% of the participants (n = 56 in each group) were prescribed to antidepressants. Because there were no differences between the two groups, it was not included in the data analysis as a variable to be controlled.

**Table 3:** Analysis of secondary outcomes at 4 and 6 months post-randomization.

Analysis	Therapist-guided cCBT intervention			Enhanced usual care intervention			Adjusted difference in means (95% CI)^a^		*P* Value	
	Baseline	4 mo	6 mo	Baseline	4 mo	6 mo	4 mo	6 mo	4 mo	6 mo
CATS	19.5 (8.6)	9.8 (8.4)	9.5 (9.0)	20.5 (9.5)	14.0 (9.8)	11.9 (9.6)	–3.76	–1.81	0.002	0.168
	(n = 108)	(n = 94)	(n = 80)	(n = 108)	(n = 95)	(n = 82)	(–6.15 to -1.37)	(–4.40 to 0.77)		
SPSI-RS	9.5 (2.6)	11.6 (3.0)	11.5 (3.1)	9.2 (2.4)	10.5 (2.7)	10.3 (2.7)	1.01	1.00	0.009	0.025
	(n = 108)	(n = 90)	(n = 80)	(n = 107)	(n = 92)	(n = 79)	(0.26 to 1.77)	(0.13 to 1.89)		
Physical well-being	30.7 (7.5)	36.3 (9.0)	36.2 (7.8)	30.0 (7.1)	33.8 (8.2)	34.9 (9.10)	2.67	1.37	0.024	0.292
	(n = 108)	(n = 93)	(n = 78)	(n = 108)	(n = 92)	(n = 82)	(0.35 to 4.99)	–1.19 to 3.94)		
Psychological well-being	30.9 (6.7)	42.9 (12.7)	42.2 (12.7)	29.6 (6.7)	38.6 (10.3)	39.6 (10.5)	4.02	2.51	0.018	0.159
	(n = 107)	(n = 94)	(n = 78)	(108)	(n = 93)	(n = 83)	(0.70 to 7.34)	(–1.00 to 6.02)		
Autonomy and parent relation	40.3 (8.9)	43.6 (12.0)	43.8 (12.1)	37.8 (10.1)	42.0 (9.6)	41.3 (10.4)	0.52	–0.28	0.677	0.842
	(n = 108)	(n = 94)	(n = 78)	(n = 108)	(n = 92)	(n = 82)	(–1.93 to 2.96)	(–3.10 to 2.53)		
Peers and social support	42.2 (13.5)	46.5 (13.3)	46.7 (15.5)	43.0 (13.1)	47.1 (12.5)	47.6 (13.5)	–0.68	–0.04	0.684	0.983
	(n = 108)	(n = 94)	(n = 78)	(n = 108)	(n = 94)	(n = 83)	(–3.97 to 2.61)	(–4.11 to 4.02)		
School environment	36.6 (9.1)	44.6 (10.4)	47.0 (10.5)	36.8 (8.9)	43.5 (11.2)	42.7 (11.8)	2.13	4.61	0.164	0.009
	(n = 105)	(n = 83)	(n = 66)	(n = 104)	(n = 83)	(n = 69)	(–0.88 to 5.15)	(1.20 to 8.02)		
General HRQoL Index	33.8 (6.1)	41.9 (10.1)	41.6 (9.6)	32.3 (6.7)	39.3 (7.6)	39.8 (7.9)	2.56	1.43	0.053	0.309
	(n = 104)	(n = 83)	(n = 66)	(n = 104)	(n = 83)	(n = 69)	(–0.03 to 5.15)	(–1.35 to 4.22)		

*CATS, Children’s Automatic Thoughts Scale; cCBT: computer-assisted cognitive-behavioral therapy; SPSI-RS, Social Problem Solving-Inventory – Revised Short Form; HRQoL, Health-Related Quality of Life; mo, months.*

Data are mean (standard deviation), unless otherwise specified.

a Outcome adjusted for sex and baseline scores.

### Additional Analysis

#### Compliance and Satisfaction With Treatment

The mean number of therapist-guided cCBT intervention sessions attended was 5.38 (SD 2.7), and treatment compliance was found in 63.9% of the patients. Adolescents in the experimental treatment group were significantly more satisfied with treatment, with the PHC centers’ facilities, with the psychological care received, and with non-professional staff than those in the comparator treatment group. There were no differences between both arms in terms of satisfaction with the medical care received (see **Table 4**).

Assessments of the fidelity of the experimental treatment delivery revealed that sessions lasted a mean of 30.6 minutes (SD 9.7, range 16 to 59), and that the therapists delivered 94.7% of the proposed activities with good quality (SD 8.4, range 70 to 100).

**Table 4:** Satisfaction with treatment at 6 months post-randomization.

	No. (%)		
	Therapist-guided cCBT intervention n = 108	Enhanced usual care intervention n = 108	*P* Value
Satisfaction with treatment, [n], mean, (SD)	[n = 84] 6.1 (1.1)	[n = 86] 5.4 (1.6)	0.002
Satisfaction with facilities, [n], mean, (SD)	[n = 82] 6.1 (0.1)	[n = 89] 5.6 (0.2)	0.012
Satisfaction with medical care, [n], mean, (SD)	[n = 81] 6.6 (0.9)	[n = 89] 6.4 (0.1)	0.116
Satisfaction with psychological care, [n], mean, (SD)	[n = 81] 6.8 (0.1)	[n = 86] 6.2 (0.2)	0.003
Satisfaction with non-professional staff treatment, [n], mean, (SD)	[n = 82] 6.5 (0.1)	[n = 89] 6.0 (0.1)	0.010

*cCBT, computer-assisted cognitive behavioral therapy; No, frequency; SD, standard deviation.*

[n] = Number of individuals when it does not match the initial number of 108 subjects per group.

#### Interaction Between Randomization Arm and Baseline Depression Severity.

There was no evidence that the effect of the intervention on the BDI score at 4 months was modified by baseline depression severity (mild symptomatology was used as reference group). For moderate symptomatology, the interaction coefficient was -1.49 (95% CI, −7.98 to 5.00; P = .651), and for severe symptomatology, the interaction coefficient was -3.83 (95% CI, -10.47 to 2.81; P = 0.257). There was no evidence that the effect of the intervention on the BDI score at 6 months was modified by baseline depression severity (mild symptomatology was used as reference). For moderate symptomatology, the interaction coefficient was -1.24 (95% CI, −8.21 to 5.73; P = .7261), and for severe symptomatology, the interaction coefficient was -2.18 (95% CI, -9.25 to 4.90; P = 0.544). Therefore, the interactions terms were not included in the analyses of the primary outcome.

#### Remission Rates

Remission rates were significantly higher in the experimental treatment group at 4 months post-randomization [40.0% *vs*. 26.5%, adjusted odds ratio (OR) = 1.95, 95% CI 1.04 to 3.65, P = 0.036], and higher but not statistically significant at 6 months post-randomization (43.7% *vs*. 36.3, adjusted OR = 1.31, 95% CI 0.69 to 2.49, P = 0.414).

## Discussion

### Main Results and Importance

A brief therapist-guided cCBT intervention was found to be superior to the EUC for adolescent depression with respect to measures of depressive symptoms, dysfunctional thoughts, social problem-solving abilities, perception of school environment, physical and psychological well-being, general HRQoL, and remission rates of depression. Moreover, adolescents receiving this experimental treatment were more satisfied with the psychological intervention, *versus* those assigned to the comparator treatment. Importantly, considering that the comparator condition was an active treatment and that this study was carried out in PHC settings, the primary outcome effect size (*d* = 0.39) was higher than the average effect size reported in similar studies that used active control groups (*d* = 0.24) or were conducted in clinical service settings (*d* = 0.24) (53). However, apart from social-problem abilities and perception of school environment, statistically significant treatment gains were not maintained over time for the study outcomes.

This study is the first to report evidence on the efficacy of a brief therapist-guided cCBT intervention for adolescent depression in Latin America. As this trial was carried out in real-life PHC settings serving an urban poor population in one of the largest cities of the Region, it valuably contributes to reducing the gap in scientific knowledge regarding the treatment of depression in vulnerable groups receiving mental health care attention in resource-constrained settings (54). Furthermore, the attractiveness of a therapist-guided computer-assisted psychotherapy may have contributed to the treatment adherence in a socio-economically deprived sample of adolescents with previous mental health problems (24). Likewise, this experimental treatment demonstrates the potential of technology-assisted interventions as a driver for the availability and uptake of evidence-based mental health practices in general health services.

### Comparison With Previous Work

A recent meta-analysis on the effects of integrated medical-behavioral care (i.e., provision of mental health care in PHC) for child and adolescent mental health outcomes revealed a medium and significant effect size for improved youth outcomes (*d* = 0.42) (27), much in line with the one reported in this trial (*d* = 0.39). Although far from the Cohen’s *d* values of 0.63 found for collaborative care models (27), it must be borne in mind that these latter interventions require important changes in the provision of mental health care, representing complex multi-component interventions compared to the stand-alone experimental treatment described in the present study. In the future, the demonstrated efficacy of a therapist-guided cCBT intervention may be reinforced by organizational changes (i.e., integrated into a multi-component collaborative care model for adolescent depression) to support the integration of adolescent mental health care in PHC (27), thus providing significant and sustainable treatment effects to depressed adolescents, especially in the case of those with severe symptoms of depression.

The feasibility and acceptability of technology-assisted interventions (whether computer-based or not) for depression are well established (55, 56); however, most of clinical trials have been carried out in adult populations, and the evidence on computer-assisted psychotherapies for adolescent depression remains scarce. When cCBT for youth depression has been applied in school and health services, its effectiveness and acceptability among users have been demonstrated (25, 26). The results of this study extend this evidence to the use of technology to facilitate the necessary integration of adolescent mental health services in PHC centers. Moreover, though some interventions have been developed for use in specialized mental health services (57) or in clinical contexts (58), these programs have not been integrated into the usual practice of PHC centers.

For example, in the pilot RCT by Stallard et al. (57), mental health services recruited patients, but the intervention was provided in participants’ homes, by a trained clinician. Subsequently, the same cCBT program was evaluated in school health contexts, with promising results (59). Merry et al. (58), for their part, tested the effectiveness of a self-help software package *versus* usual treatment in PHC and school health services, demonstrating its non-inferiority. The only evidence of a program integrated into clinical practice comes from a pilot clinical trial in the US that explored the implementation of an iPad-assisted CBT program (60). The feasibility of the program was demonstrated for depression among adolescents, achieving higher user satisfaction, but it was not superior than treatment as usual with respect to reductions of depressive symptoms (60).

### Limitations

The promising results of this RCT must be considered along with its limitations. First, as the sample size was calculated to detect differences at 4 months post-randomization, it may have lacked enough statistical power to detect potentially meaningful effects at 6 months post-randomization. Second, although a RCT is often referred to as the gold standard of clinical research, the non-blinding of participants (i.e., patients and providers) along with the use of patient self-reported outcomes may have increased the risk of bias and produced an over estimation of the potential benefits of the intervention. Third, in addition to the high non-compliance rate among the patients in the experimental treatment group, weekly sessions had to be rescheduled due to barriers in PHC centers or participants (e.g., illness, holidays, study obligations), producing alterations in the periodicity of treatment. Fourth, as currently the Chilean Ministry of Health Clinical Guidelines for the Management of Depression do not specify the number of sessions for psychosocial interventions, nor its periodicity, adherence to guidelines in the comparator treatment group was not assessed. Fifth, the use of psychologists may prevent generalizability to other low-resource settings. It is worth clarifying that, in Chile, psychologists are in charge to provide psychotherapy in PHC centers. However, many of them do not have specific training or certification in psychotherapy. Sixth, data such as indicated dose or adherence to the pharmacological treatment was not collected. However, in both groups, the pharmacological treatment was in charge of the same medical doctors of the PHC centers, and there were no differences in the proportion of participants who received medication. Finally, the therapist-guided cCBT intervention relied almost exclusively on the use of patient-level (i.e., individual) strategies; this may have been one of the reasons for the lack of an apparent effect on the HRQoL subscales of peers and social support, and parent relationships. Thus, a future therapist-guided cCBT intervention must consider these limitations, having sufficient statistical power to detect significant differences at the last follow-up assessment, including clinician-reported outcomes, testing new strategies to improve adherence to and periodicity of treatments, and including the use of therapeutic strategies at the family or school level.

### Take Home Message

The feasibility, acceptability, and proven efficacy of a brief therapist-guided cCBT intervention described in this study have relevant implications for the treatment of adolescent depression in PHC settings. Most depressed adolescents who require care do not obtain it, and those who manage to access help often do not show clinical improvements, possibly because the psychological treatment received is not sufficiently tailored to their needs. Among other strengths, the use of cCBT helps to ensure an improved fidelity with evidence-based treatments. Improvements were found for most of the therapeutic skills that were taught, an outcome that was not found with a universal intervention of a similar program (44). The loss of clinical gains with time has been commonly described in the literature on psychological treatments for depressed adolescents (17). Future studies should investigate whether the addition of post-treatment booster sessions can help to maintain the benefits initially obtained, and whether relying more on technologies (e.g., blending face-to-face cCBT with a low-intensity Internet-based intervention between treatment sessions) would reinforce treatment effects and their sustainability (61). Furthermore, as the therapist-guided cCBT intervention reported in this study was provided by study psychologists, forthcoming versions of the experimental treatment should test whether it is feasible for PHC psychologists to deliver these types of service. Once ensured that the clinical gains are sustained over time, cost-effectiveness evaluations of an intervention of these characteristics will be needed to support the evidence in favor of the dissemination and scale-up of cCBT treatments for depression in PHC centers.

### Conclusions

A brief therapist-guided cCBT eight-session intervention improved behavioral health outcomes of depressed adolescents treated in PHC centers of a Latin American developing country. However, differences of between groups were not sustained over time. This trial importantly contributes to reduce the gap in scientific knowledge with respect to treatment of depression in vulnerable groups of the population and provides one of the first examples of a technology-assisted intervention to integrate adolescent depression treatment into PHC. Future research should focus on exploring new strategies to sustain and increase response, especially among adolescents with severe depression and on investigating the cost-effectiveness of this intervention before advocating for a major scale up in PHC services.

## Ethics Statement

The study was approved by the Ethic Committee of Human Research of the Faculty of Medicine of Universidad de Chile (project number 032-2012). Written informed consent was obtained directly from each participant over 18 years of age, and written informed assent and consent was taken from the underage participants and their parents (or guardians), respectively.

## Author Contributions

VM was the principal investigator of this study. VM, GR, and RA conceived the study and were involved in managing and advising the project. PM and PV contributed to the development of the project. JG and PZ performed the statistical analyses. All authors contributed to the drafting of this paper and approved the final manuscript.

## Funding

This project was funded by the Chilean National Fund for Scientific and Technological Development, FONDECYT (project number 11121637), Millennium Science Initiative of the Ministry of Economy, Development and Tourism, grant “Millennium Nucleus to Improve the Mental Health of Adolescents and Youths, Imhay,” and the Innovation Fund for Competitiveness [Fondo de Innovación para la Competitividad, FIC], part of the Chilean Ministry of Economy, Development, and Tourism, through the Millennium Scientific Initiative, Project IS130005. The funding sources had no influence on study design; the collection, analysis, and interpretation of data; the writing of the report; or the decision to submit the manuscript for publication.

## Conflict of Interest Statement

The authors declare that the research was conducted in the absence of any commercial or financial relationships that could be construed as a potential conflict of interest.
